# Functional Reorganization of Cortical Language Function in Glioma Patients—A Preliminary Study

**DOI:** 10.3389/fonc.2019.00446

**Published:** 2019-05-29

**Authors:** Sebastian Ille, Lara Engel, Lucia Albers, Axel Schroeder, Anna Kelm, Bernhard Meyer, Sandro M. Krieg

**Affiliations:** ^1^Department of Neurosurgery, Klinikum rechts der Isar, School of Medicine, Technische Universität München, Munich, Germany; ^2^TUM-Neuroimaging Center, Technische Universität München, Munich, Germany

**Keywords:** functional reorganization, glioma, language, nrTMS, plasticity

## Abstract

**Background:** Functional reorganization (FR) was shown in glioma patients by direct electrical stimulation (DES) during awake craniotomy. This option for repeated mapping is available in cases of tumor recurrence and after decision for a second surgery. Navigated repetitive transcranial magnetic stimulation (nrTMS) has shown a high correlation with results of DES during awake craniotomy for language-negative sites (LNS) and allows for a non-invasive evaluation of language function. This preliminary study aims to examine FR in glioma patients by nrTMS.

**Methods:** A cohort of eighteen patients with left-sided perisylvian gliomas underwent preoperative nrTMS language mapping twice. The mean time between mappings was 17 ± 12 months. The cortex was separated into anterior and posterior language-eloquent regions. We defined a tumor area and an area without tumor (WOT). Error rates (ER = number of errors per number of stimulations) and hemispheric dominance ratios (HDR) were calculated as the quotient of the left- and right-sided ER.

**Results:** In cases in which most language function was located near the tumor during the first mapping, we found significantly more LNS in the tumor area during the second mapping as compared to cases in which function was not located near the tumor (*p* = 0.049). Patients with seizures showed fewer LNS during the second mapping. We found more changes of cortical language function in patients with a follow-up time of more than 13 months and lower WHO-graded tumors.

**Conclusion:** Present results confirm that nrTMS can show FR of LNS in glioma patients. Its extent, clinical impact and correlation with DES requires further evaluation but could have a considerable impact in neuro-oncology.

## Introduction

For low- (LGG) and high-grade gliomas (HGG), a maximum extent of resection (EOR) is the crucial step for an optimal treatment (1, 2). Furthermore, recently published long-term outcome data showed us the prolonged impact of supra-total resections on the outcome of patients suffering from LGG (3). However, gliomas sometimes recur and transform to a higher grade and then require a re-resection (4). During the resection after the initial diagnosis and when re-resections are pending, functionality must be preserved, most of all by the gold standard technique of direct electrical stimulation during awake surgery (DES) (5). As we have learned from DES data, particularly in cases of language mappings during repeated awake craniotomies, cortical brain function reorganizes over time (6–10).

Apart from DES, various non-invasive mapping techniques have been used to examine FR, such as functional magnetic resonance imaging (fMRI), magnetoencephalography (MEG) and navigated transcranial magnetic stimulation (nTMS). In a recently published study, we were able to show that nTMS can measure FR of cortical motor representations (11). Regarding its underlying mechanisms, navigated repetitive TMS (nrTMS) used for language mapping is similar to the intraoperative mapping by DES (12). Former comparisons of these techniques have shown that pre- (nrTMS) and intraoperative (DES during awake craniotomy) language mapping results correlate accurately, particularly for language-negative sites (LNS), as shown by their high sensitivity and negative predictive value (13–16). Therefore, in contrast to previous studies investigating FR of language invasively by DES, this first study on non-invasive language mapping uses changes in LNS to evaluate FR.

The present study therefore aims to examine if we can measure cortical FR of language functions by nrTMS in patients suffering from language-eloquent gliomas. Furthermore, the study evaluates the ability to predict patterns of FR and analyzes various stimuli and conditions that might lead to FR of cortical language function.

## Methods

### Ethics

The experimental setup was approved by our local ethics committee (registration number: 222/14), and the experiment was conducted in accordance with the Declaration of Helsinki. Written informed consent was obtained from all patients prior to the examination and patients gave written informed consent for the publication of pseudonymized data.

### Patients

We prospectively included patients suffering from left-sided perisylvian brain lesions for our language mapping cohort who were scheduled for tumor resection at our department. For the present analysis, patients had to undergo nrTMS language mapping twice due to at least two tumor resections or a second nrTMS language mapping to evaluate a second tumor resection or as part of a standard follow-up. We performed preoperative nrTMS language mappings between 1 and 5 days before surgery. Table 1 additionally shows the time between the first and second nrTMS language mapping (Table 1). Exclusion criteria were age < 18 years; a too severe preoperative aphasia (baseline object naming < 60% of pictures in the data set properly named), making nrTMS language mapping impossible; and general TMS exclusion criteria, such as cochlear implants or a cardiac pacemaker(17).

**Table 1:** Patient characteristics.

**ID**	**Age range**	**Months between mappings**	**Seizures before PRE-1/PRE-2**	**AED**	**Language performance**					
					**PRE-1**	**POD5-1**	**POM3-1**	**PRE-2**	**POD5-2**	**POM3-2**
1	46–50	1	PRE-1	Y	1A	0	–	0	0	0
2	46–50	41	PRE-1	Y	2B	1B	1B	1B	–	–
3	41–45	12	PRE-1 and PRE-2	Y	0	1A	1A	1A	1A	1A
4	18–20	20	N	N	0	0	0	0	2A	0
5	51–55	21	N	N	2B	1B	0	0	0	2A
6	51–55	30	PRE-1	Y	0	0	0	0	–	–
7	51–55	19	PRE-2	N	2A	1A	1A	2A	2A	1A
8	21–25	11	PRE-1	Y	1A	1A	1A	1A	1A	1A
9	31–35	15	PRE-1	Y	1B	1B	0	1A	1A	1A
10	26–30	6	PRE-1	Y	0	0	0	1B	–	–
11	31–35	26	PRE-1	Y	0	1A	0	0	0	0
12	51–55	14	PRE-1 and PRE-2	Y	0	0	0	1B	1B	1B
13	46–50	37	PRE-1	Y	0	1B	0	0	0	0
14	56–60	3	N	N	0	0	0	1A	2A	1A
15	71–75	9	PRE-1	Y	0	0	0	1A	1A	1A
16	36–40	30	PRE-1	Y	0	0	0	0	–	–
17	71–75	13	PRE-1	Y	0	0	0	0	0	0
18	71–75	2	PRE-1	Y	0	2A	0	0	–	–
Mean	46	17								
SD	16	11								

*The table shows the characteristics of all included patients (SD, standard deviation; Y, yes, N, no; AED, anti-epileptic drugs; PRE-1, before first surgery, POD5-1, 5 days after first surgery; POM3-1, 3 months after first surgery; PRE-2, before second surgery/evaluation of a second surgery; POD5-2, 5 days after second surgery/evaluation of a second surgery; POM3-2, 3 months after second surgery/evaluation of a second surgery; A, non-fluent aphasia; B, fluent aphasia)*.

### Setup

All patients obtained structural MRI scans on a 3T MR scanner (Achieva 3T, Philips Medical System, Netherlands B.V.). The standard MRI protocol includes a three-dimensional (3-D) gradient echo sequence with intravenous contrast administration for anatomical coregistration and diffusion tensor imaging (DTI) sequences with 32 orthogonal sequences. Postoperatively, the same MRI scan was performed within 48 h after surgery for the EOR calculation.

We examined all patients' neurological statuses, including aphasia grading adapted from the Aachener Aphasia Test (AAT) (0 = no impairment of language function; 1 = slight impairment of daily communication; 2 = moderate impairment of language function, daily communication possible; 3 = severe impairment of language function, daily communication not possible; A = non-fluent; B = fluent), history of seizures and the Edinburgh Handedness Inventory (EHI) at no fewer than six points in time: before the first surgery (PRE-1), 5 days after the first surgery (POD5-1), 3 months after the first surgery (POM3-1), before the second surgery or in case of the evaluation of a second surgery (PRE-2), 5 days after the second surgery (POD5-2) and 3 months after the second surgery (POM3-2). Since we also included patients who already underwent tumor resections before the initial surgery as counted for the presented study, PRE-1, POD5-1, and POM3-1 indicates for the index-surgery 1 of the present study as well as PRE-2, POD5-2, and POM3-2 indicates for the index-surgery 2 of the present study. The neurological and neuropsychological status was determined in accordance of the examination two advanced neurosurgeons and a technical assistant who is well-experienced in neuropsychological assessments.

### Navigated Repetitive Transcranial Magnetic Stimulation Mapping

We used the eXimia nTMS system version 4.3 and a NEXSPEECH® module (Nexstim Plc, Helsinki, Finland) for nrTMS language mappings. We performed mappings according to our recently published nTMS working group protocol (18).

In short, we used black and white drawings of common objects without a written lead-in phrase as stimuli, i.e., our standard object naming task (ON). After baseline testing (naming without nrTMS stimulation), we discarded misnamed objects. We then presented the remaining objects time-locked to nrTMS pulses with a picture-to-trigger interval (PTI) of 0 ms while the stimulation coil was randomly moved over the whole hemisphere using the most effective stimulation frequency and intensity (5Hz/5 pulses, 7/7, or 10/10). Each of the 46 predetermined stimulation sites was stimulated three times and, if possible considering patient status and compliance, on both hemispheres with an interpicture interval (IPI) of 2,500 ms and a picture presentation time (PPT) of 700 ms. Hence, in patients who were able to undergo the mapping of both hemispheres, we stimulated 276 times in total. Afterward, we analyzed naming errors (no response, performance, hesitation, neologism, semantic, phonological, circumlocution) by comparing the baseline ON with the ON during nrTMS stimulation (18). Hesitation errors were excluded from the present analysis. Finally, the results of nrTMS language mappings were transferred to the neuronavigation system, were included in the individual decision-making process, and were displayed on the neuronavigation screen during each tumor resection according to the standard protocol at our department (19).

### FR Analysis

First, we allocated language-positive stimulation sites in terms of nrTMS and the respective language error subtype to the 46 predetermined cortical stimulation sites. Due to nrTMS's high sensitivity and negative predictive value compared to the gold standard technique DES during awake surgery in former studies, we focused on the evaluation of changes in LNS (= stimulation sites without any language error induced by nrTMS stimulation) (13–16).

We then subdivided the patients into several subgroups and performed a case-by-case analysis based on the following characteristics: WHO grade, time between mappings, language deficits, tumor location, inter- and intra-hemispheric dominance ratios, predominant localization of language function, seizures, AEDs and handedness.

### Separation of Cortex

To perform statistical analysis, we separated the cortex into anterior (triangular part of the inferior frontal gyrus, opercular part of the inferior frontal gyrus and ventral precentral gyrus) and posterior (anterior supramarginal gyrus, posterior supramarginal gyrus, middle superior temporal gyrus, posterior superior temporal gyrus and angular gyrus) language-eloquent areas. Based on this separation and the tumor location in the initial MRI scan before the first surgery, we assigned patients to two groups: patients with anterior (A) and posterior (P) tumors. Consequently, we defined a tumor area (T) and an area without tumors (WOT) for each patient. For example, if a patient suffers from a tumor within the frontal operculum, then he or she is assigned to tumor group A; A corresponds to his or her tumor area, and P corresponds to his or her area without tumor.

### Statistical Analysis

In order to analyze changes of LNS between the two nrTMS language mappings in dependence on different characteristics, we calculated *p*-values for the mean changes of LNS between mapping 1 and mapping 2, for the differences between two characteristics, and for the differences between LNS in the tumor area and in the area without tumor. A *p* < 0.05 was considered significant. For the calculation of the *p*-values we used the Wilcoxon-Test and the Mann-Whitney-U-Test. Statistical analysis was performed by the use of GraphPad Prism software (GraphPad Prism 6.04, La Jolla, CA, USA).

Apart from the LNS analysis, error rates (ER = language errors induced by nrTMS per stimulations) were also calculated to determine each patient's hemispheric dominance in terms of nrTMS. The hemispheric dominance ratio (HDR = ER left hemisphere divided by ER right hemisphere, i.e., HDR >1 left dominant, HDR < 1 right dominant) was further subdivided into an anterior HDR (aHDR = ER within left-sided A divided by ER within right-sided A) and a posterior HDR (pHDR = ER within left-sided P divided by ER within right-sided P). Furthermore, we calculated an intrahemispheric ratio (IHR; IHR L = ER within left-sided A divided by ER within left-sided P; IHR R = ER within right-sided A divided by ER within right-sided P).

## Results

### Patient Characteristics and nrTMS Language Mapping Results

Between January 2013 and September 2017, we included 18 patients who received at least two nrTMS language mappings. Second mappings were performed due to tumor recurrence and reoperation in 15 cases and as part of the standard follow-up in 3 cases. Gross total resection was performed in 16 of 18 cases (89%) during surgery 1. Histopathology showed gliomas in all cases. Tables 1, 2 show detailed patient characteristics including adjuvant treatments after surgery 1.

**Table 2:** Tumor characteristics.

**ID**	**Tumor location**	**Surgery before PRE1**	**EOR surgery 1 (%)**	**EOR surgery 2 (%)**	**Tumor**								
					**Surgery 1**				**Surgery 2**				**Adjuvant treatment after surgery 1**
					**Entity**	**WHO**	**IDH mutation**	**1p19q codeletion**	**Entity**	**WHO**	**IDH mutation**	**1p19q codeletion**	
1	P	Y	80	100	GBM	IV	Y	-	GBM	IV	Y	-	N
2	P	N	100	–	AA	III	Y	N	no surgery	–	–	–	TMZ
3	P	N	100	100	GBM	IV	N	–	GBM	IV	N	–	RT/TMZ
4	A	Y	100	70	OD	II	N	–	OD	II	N	N	N
5	P	N	100	100	GBM	IV	–	–	GBM	IV	–	–	RT/TMZ
6	A	N	90	–	AA	III	Y	N	no surgery	–	–	–	TMZ
7	A	N	100	80	GBM	IV	–	–	GBM	IV	–	–	RT/TMZ
8	A	N	100	100	DA	II	Y	N	DA	II	Y	N	N
9	A	Y	100	70	AA	III	Y	N	AA	III	Y	N	TMZ
10	P	N	100	–	GBM	IV	N	–	GBM (Biopsy)	IV	N	–	RT/TMZ
11	A	N	100	100	AA	III	Y	N	AA	III	Y	N	TMZ
12	P	Y	100	100	AA	III	N	N	GBM	IV	N	–	RT/TMZ
13	A	Y	100	100	DA	II	Y	N	DA	II	Y	N	TMZ
14	P	N	100	90	GBM	IV	N	–	GBM	IV	N	–	RT/TMZ
15	P	N	100	100	GBM	IV	N	N	GBM	IV	N	N	RT/TMZ
16	A	Y	100	–	AA	III	Y	N	no surgery	–	–	–	TMZ
17	P	N	100	100	GBM	IV	N	–	GBM	IV	N	–	RT/TMZ
18	A	N	100	80	GBM	IV	N	–	GBM	IV	N	–	RT/TMZ

*The table shows tumor characteristics of all included patients (A, anterior; P, posterior; Y, yes; N, no; EOR, extent of resection; GTR, gross total resection; STR, subtotal resection; DA, diffuse astrocytoma; AA, anaplastic astrocytoma; OD, oligodendroglioma; GBM, glioblastoma; RT, radiation therapy; TMZ, temozolomide)*.

The Supplementary Table 1 shows detailed nrTMS language mapping results of all mappings and patients as well as changes in IHR or HDR. Without showing statistical significance, we found a distinct increase of inter- and intra-hemispheric changes after 13 months, when contrasting the IHRs and HDRs arranged in order of time between mappings. These changes were more likely in patients suffering from gliomas, WHO grade II or III (Supplementary Table 1). We could not find differences of the FR of patients suffering from anterior tumors as compared to patients suffering from posterior tumors (Tables 2, 3).

**Table 3:** Language-negative stimulation sites left hemisphere.

**ID**	**No. of language-negative stimulation sites left hemisphere**					
	**Tumor area**			**Area w/o tumor**		
	**Mapping**		**More in 2nd mapping**	**Mapping**		**More in 2nd mapping**
	**1**	**2**		**1**	**2**	
1	3	6	Y	0	3	Y
2	12	1	N	2	3	Y
3	10	7	N	6	0	N
4	1	1	-	6	10	Y
5	3	7	Y	0	3	Y
6	4	1	N	9	2	N
7	0	1	Y	7	6	N
8	3	2	N	10	7	N
9	0	2	Y	1	8	Y
10	8	4	N	2	5	Y
11	3	2	N	8	13	Y
12	7	4	N	4	4	-
13	0	2	Y	7	10	Y
14	1	1	-	2	1	N
15	8	3	N	1	0	N
16	1	6	Y	5	10	Y
17	10	7	N	7	0	N
18	1	4	Y	6	7	Y

*The table shows language-negative stimulation sites within anterior and posterior language-eloquent areas of all patients and mappings (Y, yes; N, no)*.

Table 3 shows the changes from first to second mapping within the tumor area and the WOT area for all patients (Table 3). Table 4 shows overall LNS changes between the first and second mappings depending on various patient characteristics and the according statistical analysis in order to show the impact on FR. In case of most language function was located near the tumor during the first mapping, we could find more LNS within the tumor area during the second mapping. This decrease of language function in the tumor area showed a statistically significant difference to cases in which language function was not located near the tumor (*p* = 0.049; Table 4).

**Table 4:** Dependence on different characteristics.

		**Tumor area**					**Area w/o tumor**					**Tumor area vs. area w/o tumor**
		**Mapping 1**	**Mapping 2**		**Difference**		**Mapping 1**	**Mapping 2**		**Difference**		
Language near tumor	Total	26	34	*p =* 0.41	+8	*p =* 0.049	65	76	*p =* 0.50	+11	*p =* 0.36	*p =* 0.86
	Mean	2.4	3.1		0.7		5.9	6.9		1		
Language not near tumor	Total	49	27	*p =* 0.17	−22		18	16	*p >* 0.99	−2		*p =* 0.31
	Mean	7	3.9		−3.1		2.6	3.3		−0.3		
Aphasia POD5-1	Total	32	28	*p =* 0.77	−4	*p =* 0.42	47	57	*p =* 0.40	+10	*p =* 0.62	*p =* 0.77
	Mean	3.6	3.1		−0.4		5.3	6.2		1.1		
No aphasia POD5-1	Total	43	33	*p =* 0.44	−10		36	35	*p >* 0.99	−1		*p =* 0.55
	Mean	4.8	3.7		−1.1		4	3.9		−0.1		
Surgery-related deficit	Total	14	15	*p =* 0.14	1	*p =* 0.25	27	30	*p >* 0.99	3	*p =* 0.63	*p =* 0.24
	Mean	4.9	3.1		−1.7		3.3	3.4		1.4		
Tumor-related deficit	Total	45	33	*p >* 0.99	−12		35	31	*p =* 0.88	−4		*p >* 0.99
	Mean	3.5	3.8		0.3		6.8	7.5		0.8		
Seizures	Total	70	52	*p =* 0.30	−18	*p =* 0.28	75	78	*p =* 0.82	3	*p =* 0.59	*p =* 0.31
	Mean	4.7	3.5		−1.2		5	5.2		0.2		
No seizures	Total	5	9	*p* > 0.99	4		8	14	*p =* 0.50	6		*p >* 0.99
	Mean	1.7	3		1.3		2.7	4.7		2		

*The table shows overall language-negative stimulation sites (LNS) of the left hemisphere in dependence on different characteristics (POD5-1 = 5 days after first surgery)*.

### Illustrative Case

#### Patient 13

**Figure 1:**
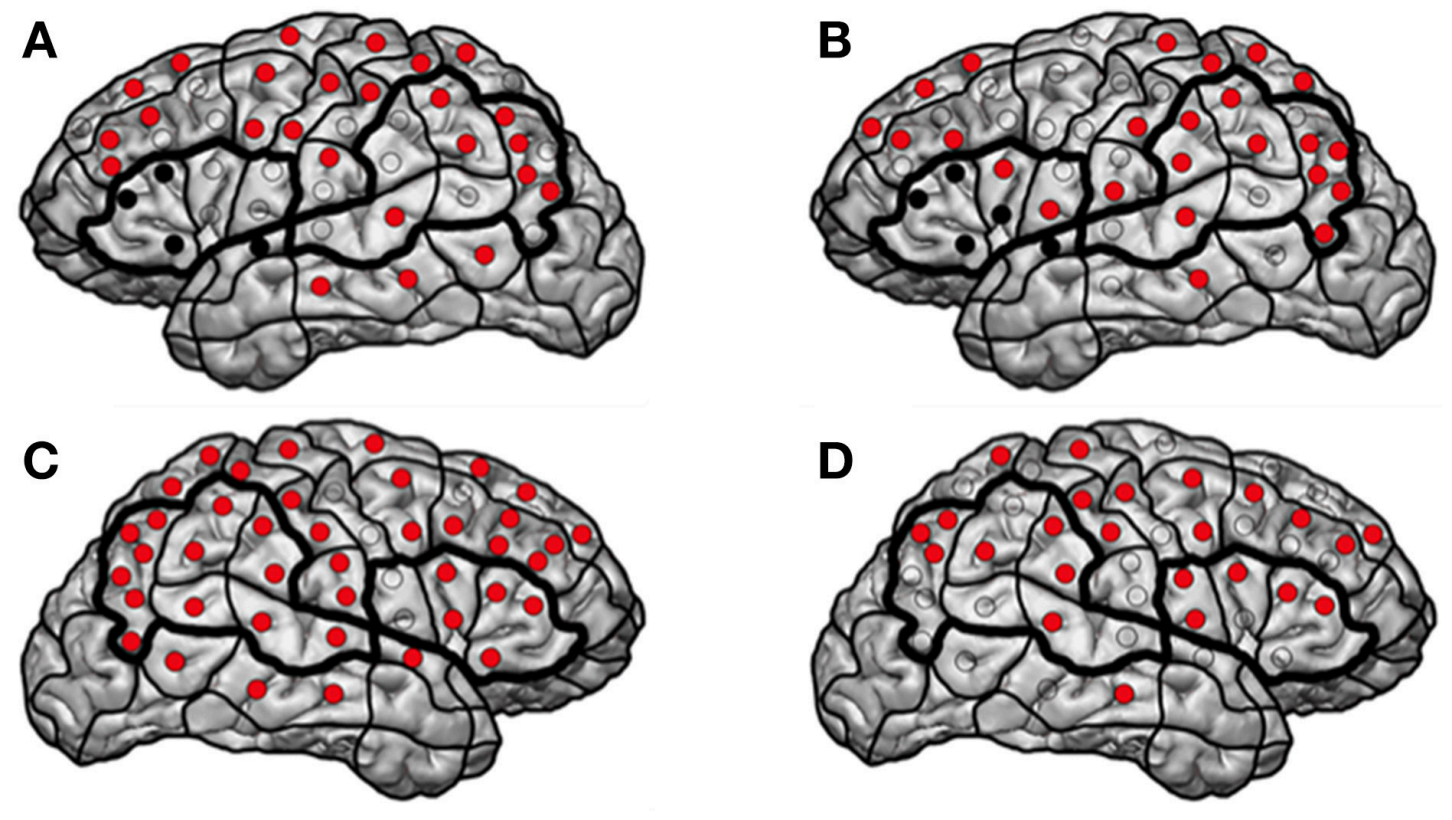
Patient 13. The figure shows the results of the first **(A,C)** and second **(B,D)** left- and right-hemispheric nrTMS language mappings of patient 13. Red sites indicate language-negative stimulation sites, and black sites indicate resected sites in terms of subgyri as a functional unit.

The patient presented at our department due to tumor recurrence of a left-sided insular diffuse LGG (DLGG), WHO grade II. The first resection was performed 6 years ago (Tables 1, 2). We performed preoperative nrTMS language mapping and intraoperative DES language mapping during awake craniotomy (Figures 1A,C). Language-positive sites could be found by DES and nrTMS within the ventral precentral gyrus (Figure 1A). According to nrTMS language mapping results, the patient had a left-hemispheric dominance with an HDR of 1.35 and an anteriorly located dominance with an IHR of 1.13 (Figures 1A,C, Supplementary Table 1). We performed a GTR, and histopathology again showed a diffuse astrocytoma, WHO grade II (IDH mutation positive, no 1p19q-co-deletion) (Table 2). The patient showed a transient fluent aphasia, grade 1B, at POD5-1 but no language deficit at POM3-1.

Three years later, a follow-up MRI scan showed a tumor recurrence. We again performed preoperative nrTMS language mapping and intraoperative DES language mapping during awake craniotomy (Figures 1B,D). LNS according to preoperative nrTMS language mapping now showed an increase of LNS in the tumor area (0 vs. 2) and the WOT area (7 vs. 10) although HDR was 1.18 and IHR was 1.71 due to high ERs at single stimulation sites. Furthermore, right-hemispheric nrTMS language mapping showed fewer LNS during the second mapping. DES language mapping did not show any cortical language-positive sites within the craniotomy area. A postoperative MRI scan again showed a GTR, and the patient did not suffer from any language deficit.

## Discussion

### Detecting FR of LNS by nrTMS

With the present results, we can confirm that nrTMS language mapping is able to measure FR of language function in patients suffering from language-eloquent gliomas. Our cohort provides highly valuable and rarely available results of cortical remappings in glioma patients. As a core result of our study, we could show with statistical significance that function moves away from the tumor if it is adjacent to the tumor in comparison to cases in which function is not located near the tumor (*p* = 0.049). A large-scale study has already shown similar results regarding the remapping of cortical functions by DES during awake craniotomy (9). Further studies and case reports also showed comparable directions of FR (6, 8, 20, 21). Recently, Picart et al. published the largest cohort of repeated language mappings by DES during awake craniotomy and analyzed two groups with different levels of plasticity. This study showed that patients with a higher level of plasticity recovered faster after the reoperation and that the EOR was significantly larger than in patients with a lower level of plasticity. The authors concluded that cortical tumors with sharp borders facilitate these more efficient mechanisms of plasticity and that tumoral invasion of white matter pathways is the main limitation of plasticity (10).

Because we used nrTMS for language mappings and remappings, we focused on the analysis of LNS due to the current reliability of nrTMS for language mapping compared to the gold standard technique DES during awake craniotomy: nrTMS showed a high sensitivity (90–100%) and negative predictive value (84–100%), while specificity (24–98%) and positive predictive value (36–75%) differed in a wide range (13–16). Current paradigms of DES language mapping during awake craniotomy show us that it is sufficient to perform a mapping of LNS after the confirmation of stimulation intensity by detecting at least 1 language-positive site (5). Regarding the present study's objective, the literature shows us that focusing on LNS is inevitable when we compare our results with those from former publications (9, 20).

### Interpretation of Data and Impact of Various Characteristics on FR

As a major part of the present study, we could show that nrTMS can measure FR of cortical language function in glioma patients. Additionally, we analyzed various characteristics of glioma patients, which might lead to different FR courses (Table 4).

In cases where most language function was located near the tumor, we found a statistically significant increase of LNS in the tumor area in comparison to cases in which function was not located near the tumor (*p* = 0.049). In contrast, we found no change of hemispheric dominance in 71% of patients with more LNS in the tumor area during the second mapping. Accordingly, we found a change of hemispheric dominance in 56% of patients whose tumor area showed fewer LNS during the second mapping (Supplementary Table 1). This observation leads to the assumption that cortical language function concentrates on few sites when it is directly influenced by an infiltrating glioma but remains widespread when it is not. Patients in the present cohort suffered from new permanent and transient surgery-related language deficits in only 1 and 4 cases, respectively. When analyzing patients with surgery-related deficits at POD5-1, we also found a positive trend of more LNS in the left hemisphere without an increase of changes in hemispheric dominance (Table 4, Supplementary Table 1). Again, this observation outlines the assumption that language function seems to concentrate on single sites when it is directly influenced.

We could also find differences between patients suffering from new surgery-related language deficits at POD5-1 and patients suffering from new tumor-related language deficits at PRE-2 (Tables 1, 4). In case of new language deficits patients received a specific follow-up treatment at rehabilitation clinics. Patients with new surgery-related deficits showed more LNS within the tumor and WOT areas and a change of hemispheric dominance in only 1 case. Patients with tumor-related language deficits showed distinctly fewer LNS in the tumor area and in the WOT area, which leads us to the assumption that FR is trying to compensate for an imminent language deficit by distribution of cortical language function (Table 4 and Supplementary Table 1). These mechanisms might be too slow for a clinical compensation in some cases, which is supported by the fact that 5 of 6 patients with tumor-related language deficits suffered from quickly growing recurrent glioblastomas. Particularly, these mechanisms cannot compensate for a surgery-related deficit. This hypothesis is supported by our finding of an increase of FR after a longer period of tumor-free survival and consequently in lower-grade gliomas (Tables 1, 2). This expectable trend showing a cut-off at 13 months is also supported by the increase of IHRs and HDRs when contrasting patients arranged in order of the time between mappings (Table 1 and Supplementary Table 1).

### Mechanisms of FR

In accordance with former publications' results, we confirmed with statistical significance that function seems to leave the tumor area under special conditions (6, 8–10, 20, 21). The underlying reasons for functional reorganization (FR) or brain plasticity might vary. In case of patients suffering from gliomas, functional deficits might induce long-term FR and acute functional compensation. The glioma's infiltrative character, its location in relation to white matter pathways or the functional impairment by seizures are further potential reasons for a functional reshaping of the human brain (6, 7, 10). In the gain and loss of function, synapses play a major role on the microscopic level during *natural plasticity* and by auto-regulation during *meta-plasticity* (22, 23). Furthermore, glia cells, which are affected most in glioma patients through their dedifferentiation, also contribute to neuronal activity modulation by controlling energy metabolism and coordinating network activity (24). Another mechanism to prevent functional deficits is the unmasking of latent connections and networks (25, 26). Intra-cortical connections between pyramidal cells, which are usually inhibited, become functional through disinhibition, thereby enabling the transformation of silent synapses to functional synapses, and might be the core mechanism of short-term plasticity (27, 28).

An intrinsic reorganization within eloquent cortical areas additionally contributes to the compensation for imminent functional deficits. So-called *functional redundancies*, i.e., several representations of the same function in one area, can compensate for lesions by recruiting redundant areas and generating a local hyperexcitability (29). In the case of excessive lesions, the reorganization of functional networks entails several stages (25, 26). After the compensation by perilesional areas, regions in the same hemisphere prevent deficits, and in the case of insufficiency, homologous regions on the contralateral hemisphere seem to be recruited by the suppression of transcallosal inhibition (30). We also observed this phenomenon in our cohort as shown by the changes in intra- and inter-hemispheric dominance ratios. Here, it must be highlighted that our present results confirm the shift of language function to the right hemisphere as measured by nrTMS in two former studies (31, 32).

### Limitations

The fact that we included six patients who already underwent tumor resection before PRE-1 must be seen as a limitation of the present study. The impact of the initial tumor resection might already have induced FR before the first nrTMS language mapping of study period was performed. Furthermore, in 15 of 18 cases (83%) the second nrTMS language mapping was performed due to a reoperation because of tumor recurrence. Hence, it must be highlighted that the detected FR might have been occurred anyway on the one hand, but also due to tumor growth on the other hand. That this question cannot be answer by the present results must also be seen as a limitation of the present study.

The number of patients might also be a limitation of our study. Although the examination of FR must rely on case-by-case observations, our results have to be confirmed by a larger cohort to perform a more detailed analysis concerning predictive factors. The small cohort size and consequently small subgroups might also be a reason for the lack statistical significance in most subgroups. However, with this in mind, the statistical significance of the core result of our study additionally outlines the validity of our results.

## Conclusions

The present results show that nrTMS language mapping might be able to evaluate FR and cortical plasticity in patients suffering from highly language-eloquent gliomas. We could show with statistical significance that function seems to leave the tumor area. Apart from the initial location of language function, arising surgery- or tumor-related functional deficits, the occurrence of seizures and the tumor grade seem to influence this phenomenon.

## Ethics Statement

The experimental setup was approved by our local ethics committee (registration number: 222/14), and the experiment was conducted in accordance with the Declaration of Helsinki. Written informed consent was obtained from all patients prior to the examination. Ethikkommission der Fakultät für Medizin der Technischen Universität München, Grillparzerstraße 16, 3. Stock, Raum 3.46, 81675 München, ethikkommission@mri.tum.de +49 089 4140-4199.

## Author Contributions

SI is responsible for data acquisition and handled the acquired data, performed statistical analyses, performed literature research, and drafted the manuscript. LE was responsible for data acquisition. LA performed statistical analyses. AS and AK was responsible for data acquisition. BM approved and corrected the final version of the manuscript. SK revised the manuscript, approved and corrected the final version, and is responsible for the original idea, the concept, design, data acquisition, and statistical analyses. All authors read and approved the final manuscript.

### Conflict of Interest Statement

BM received honoraria, consulting fees, and research grants from Medtronic (Meerbusch, Germany), Icotec ag (Altstätten, Switzerland), and Relievant Medsystems Inc., (Sunnyvale, CA, USA), honoraria, and research grants from Ulrich Medical (Ulm, Germany), honoraria and consulting fees from Spineart Deutschland GmbH (Frankfurt, Germany) and DePuy Synthes (West Chester, PA, USA), and royalties from Spineart Deutschland GmbH (Frankfurt, Germany). SK is consultant for Nexstim Plc (Helsinki, Finland) and Spineart Deutschland GmbH (Frankfurt, Germany), and received honoraria from Medtronic (Meerbusch, Germany) and Carl Zeiss Meditec (Oberkochen, Germany). SK and BM received research grants and are consultants for Brainlab AG (Munich, Germany). The remaining authors declare that the research was conducted in the absence of any commercial or financial relationships that could be construed as a potential conflict of interest.

## Abbreviations

AAT: Aachener Aphasia Test
DES: Direct electrical stimulation
EHI: Edinburgh Handedness Inventory
ER: Error rate
FR: Functional reorganization
HDR: Hemispheric dominance ratio
IHR: Intrahemispheric ratio
IPI: Interpicture interval
LNS: Language-negative sites
nrTMS: Navigated repetitive transcranial magnetic stimulation
ON: Object naming task
POD5-1/-2: Five days after surgery 1/2
POM3-1/-2: Three months after surgery 1/2
PPT: Picture presentation time
PRE-1/-2: Before surgery 1/2
PTI: Picture-to-trigger interval
WOT: Without tumor area.
