# Supplementary material for: Functional Reorganization of Cortical Language Function in Glioma Patients—A Preliminary Study
Source: Front Oncol. 2019 May 29;9:446. doi: 10.3389/fonc.2019.00446 (PMC6558431; doi:10.3389/fonc.2019.00446)
Supplement: Supplementary file 1 [file Table_1.docx]

| ID | First mappings | | | | | | | | Second mappings | | | | | | | | Change of | | | | |
| --- | --- | --- | --- | --- | --- | --- | --- | --- | --- | --- | --- | --- | --- | --- | --- | --- | --- | --- | --- | --- | --- |
|  | EHI | Error rates | | Inter-hemispheric | | | Intra-hemispheric | | EHI | Error rates | | Inter-hemispheric | | | Intra-hemispheric | | HDR | aHDR | pHDR | IHR | |
|  |  | ER-L | ER-R | HDR | aHDR | pHDR | IHR-L | IHR-R |  | ER-L | ER-R | HDR | aHDR | pHDR | IHR-L | IHR-R |  |  |  | L | R |
| 1 | 100 | 0.22 | 0.15 | 1.49 | 1.16 | 2.86 | 0.95 | 2.33 | 100 | 0.31 | 0.18 | 1.75 | 1.57 | 2.92 | 2.23 | 4.13 | N | N | N | Y | N |
| 2 | 80 | 0.10 | 0.11 | 0.89 | 0.55 | 0.64 | 1.54 | 1.80 | 90 | 0.23 | 0.12 | 1.95 | 1.88 | 2.00 | 1.00 | 1.07 | Y | Y | Y | Y | N |
| 3 | 100 | 0.08 | 0.05 | 1.67 | 1.00 | 0.53 | 0.43 | 0.23 | 100 | 0.31 | 0.23 | 1.34 | 0.91 | 0.97 | 1.01 | 1.08 | N | Y | N | Y | Y |
| 4 | 60 | 0.19 | 0.14 | 1.42 | 0.89 | 3.67 | 1.81 | 7.43 | 60 | 0.05 | 0.05 | 0.93 | 3.14 | 2.70 | 1.74 | 1.50 | Y | N | N | N | N |
| 5 | 86.7 | 0.27 | 0.21 | 1.27 | 1.60 | 1.12 | 1.66 | 1.16 | 86.7 | 0.10 | 0.11 | 0.88 | 1.02 | 1.57 | 1.04 | 1.59 | Y | N | N | N | N |
| 6 | 100 | 0.14 | 0.11 | 1.26 | 1.43 | 2.68 | 1.26 | 2.35 | 100 | 0.44 | 0.48 | 0.91 | 0.73 | 0.82 | 1.29 | 1.44 | Y | Y | Y | N | N |
| 7 | 100 | 0.17 | 0.12 | 1.42 | 1.26 | 1.41 | 1.52 | 1.26 | 100 | 0.21 | 0.21 | 1.04 | 0.89 | 1.24 | 2.17 | 3.04 | N | Y | N | N | N |
| 8 | 81.1 | 0.14 | 0.13 | 1.04 | 1.04 | 0.64 | 1.45 | 0.00 | 81.1 | 0.19 | 0.21 | 0.91 | 3.91 | 0.54 | 1.20 | 0.16 | Y | N | N | N | N |
| 9 | 30 | 0.20 | n.p. | - | - | - | 2.22 | - | 100 | 0.10 | 0.10 | 1.09 | 6.56 | 0.71 | 2.48 | 0.27 | - | - | - | N | - |
| 10 | 100 | 0.16 | 0.17 | 0.94 | 1.02 | 1.11 | 1.14 | 1.23 | 100 | 0.18 | 0.22 | 0.83 | 0.14 | 1.43 | 0.18 | 1.90 | N | Y | N | Y | N |
| 11 | -80 | 0.15 | 0.08 | 1.75 | 1.76 | 2.40 | 1.48 | 2.02 | 60 | 0.02 | 0.02 | 1.00 | 2.86 | 0.49 | 5.72 | 0.98 | Y | N | Y | N | Y |
| 12 | 100 | 0.11 | 0.09 | 1.26 | 1.67 | 0.86 | 1.24 | 0.64 | 70 | 0.10 | 0.11 | 0.96 | 1.38 | 1.04 | 1.13 | 0.84 | Y | N | Y | N | N |
| 13 | 100 | 0.23 | 0.17 | 1.35 | 1.31 | 1.82 | 1.13 | 1.57 | 100 | 0.14 | 0.12 | 1.18 | 3.00 | 0.77 | 1.71 | 0.44 | N | N | Y | N | Y |
| 14 | 56 | 0.25 | 0.31 | 0.79 | 0.63 | 0.77 | 1.06 | 1.30 | 56 | 0.27 | 0.27 | 1.00 | 1.55 | 0.93 | 0.89 | 0.53 | Y | Y | N | Y | Y |
| 15 | 64 | 0.19 | 0.13 | 1.39 | 1.56 | 1.10 | 3.95 | 2.79 | 60 | 0.45 | n.p. | - | - | - | 1.31 | - | - | - | - | N | - |
| 16 | 20 | 0.29 | 0.27 | 1.06 | 1.30 | 1.04 | 1.90 | 1.52 | 40 | 0.05 | 0.03 | 1.81 | 1.19 | 1.81 | 0.78 | 1.19 | N | N | Y | Y | N |
| 17 | 40 | 0.05 | 0.06 | 0.97 | 2.28 | 0.42 | 2.30 | 0.42 | 20 | 0.17 | 0.18 | 0.94 | 1.64 | 1.11 | 2.45 | 1.67 | N | N | N | N | Y |
| 18 | 90 | 0.27 | 0.22 | 1.18 | 2.33 | 0.74 | 1.59 | 0.51 | 90 | 0.36 | 0.29 | 1.22 | 1.17 | 0.94 | 0.99 | 0.80 | N | N | Y | Y | N |
| Mean | 68 | 0.18 | 0.15 | 1.24 | 1.34 | 1.40 | 1.59 | 1.68 | 79 | 0.21 | 0.17 | 1.16 | 1.97 | 1.29 | 1.63 | 1.33 |  |  |  |  |  |
| SD | 44 | 0.07 | 0.07 | 0.26 | 0.48 | 0.92 | 0.72 | 1.63 | 24 | 0.13 | 0.11 | 0.34 | 1.50 | 0.69 | 1.16 | 0.97 |  |  |  |  |  |

**Supplementary File Table 1: Results of nrTMS language mappings**

The table shows the Edinburgh Handedness Inventory (EHI) results, error rates (ER), inter- (HDR) and intra-hemispheric (IHR) dominance ratios of all nrTMS language mappings as well as changes of ratios (L = left, R = right, a = anterior, p = posterior; Y = yes, N = no; n.p. = not performed).
